# Preparation and Characterization of Natural Deep Eutectic Solvents (NADESs): Application in the Extraction of Phenolic Compounds from Araza Pulp (*Eugenia stipitata*)

**DOI:** 10.3390/foods13131983

**Published:** 2024-06-24

**Authors:** Yasmin Caroline Nóvoa Sakurai, Ianê Valente Pires, Nelson Rosa Ferreira, Sanclayton Geraldo Carneiro Moreira, Luiza Helena Meller da Silva, Antonio Manoel da Cruz Rodrigues

**Affiliations:** 1 Programa de Pós-Graduação em Ciência e Tecnologia de Alimentos, Universidade Federal do Pará, Rua Augusto Correa S/N, Guamá, Belém 66075-900, PA, Brazil; yasmin_novoa@hotmail.com (Y.C.N.S.); iane_valente@hotmail.com (I.V.P.); nelson.ufpa@gmail.com (N.R.F.); amcr@ufpa.br (A.M.d.C.R.); 2 Instituto de Ciências Exatas e Naturais (ICEN), Universidade Federal do Pará, Rua Augusto Correa S/N, Guamá, Belém 66075-900, PA, Brazil; sanclay@ufpa.br

**Keywords:** environmentally friendly technologies, *Eugenia stipitata*, NADESs, ultrasonic pit

## Abstract

Natural deep eutectic solvents (NADESs) of choline chloride (ChCl) and fructose, glucose, citric and malic acid with different water concentration were prepared and characterized. The pH ranged from 2.34 to 7.38. An increase in the intensity at 3300 cm^−1^ (FT-IR), by the interaction between the receptor and the hydrogen donor, occurred. The water content increased the intensity in the OH region without changing the vibrational mode. The same behavior occurred in Raman spectra. NADES without water showed a high density (1.234 to 1.375 g/mL) and viscosity (0.09991 to 0.46921 Pa·s). NADESs with 20% and 40% water were selected for extracting phenolic compounds from araza (*Eugenia stipitata*), using an ultrasonic system of bath, tip, and ethanol. Araza ethanol extract had a TPC (total phenolic compounds) of 325.19 mg GAE/g, and DPPH and ABTS of 12.00 and 291.31 µmolL^−1^ Trolox g^−1^. ChCl:citric acid (1:1) containing 40% water was the most efficient NADES in the tip ultrasound extraction: a TPC of 273.40 mg GAE/g; DPPH and ABTS of 31.55 and 204.9 µmolL^−1^ Trolox g^−1^; and an extraction yield of 84% related to ethanol. NADESs as solvents can be used directly by the food industry as a final product or ingredient, without purification, and proved to be versatile, with different properties.

## 1. Introduction

Araza, also known as “yogurt fruit”, is a fruit that originates from the Amazon region and can also be cultivated in tropical and humid climate areas of Central and South America. Scientifically named *Eugenia stipitata*, this fruit is notably acidic and is most commonly consumed in juices, ice creams, and various desserts. The name of the fruit is of Tupi origin and means “plant that has eyes”, a characteristic that can be seen in the skin that gives the fruit the appearance of an eye. The araza is round and weighs between 30 g and 300 g. It has a very thin yellow skin and contains around four to ten seeds, each about 1 cm in length. In addition to the more traditional araza, there are other types, which can vary in terms of the color, size, and height of the tree. Among them, the most common are “araçá vermelho”, “araçá-de-coroa”, “araçá-da-praia”, “araçá-do-campo”, “araçá-do-mato”, “araçá-pêra”, “araçá-rosa”, and “araçá-piranga”. The best-known araza variety, the yellow araza (*cattleyanum*), is a small fruit tree native mainly to coastal sandbanks in Brazil.

Bioactive compounds have been studied in different raw materials from the Amazon biome. Among them is the araza (*Eugenia stipitata*), a fruit species with a characteristic flavor much appreciated by local consumers, which has relevant social and economic importance [1]. This fruit is rounded or flat, light yellow, very acidic, and has fleshy flesh. The araza fruit can be considered a good source of vitamin C, fibers, minerals, and compounds of pharmacological interest, such as carotenoids, terpenes, phenolic acids, and flavonoids, having a high antioxidant capacity. This fruit is usually sold fresh and consumed as juice, cocktails, nectar, jam, confectionery, and several other products. Its flavor is much appreciated in the food industry [2]. The few investigations about this fruit suggest nutritional and functional potential [3]. In a recent study, high contents of glycosylated quercetin derivatives were found in the fruit, and their potency as inhibitors of enzymes of carbohydrate metabolism seem to correspond to the pattern of glycosylation [4].

The extraction of phytochemicals with bioactive properties has aroused great interest in research, which is attributed to their health-promoting benefits [5], such as anti-inflammatory [6], antidiabetic [7], anti-obesity [8], antiatherosclerotic [9], antimicrobial [6], antitumor [10], antihypertensive [11], and antioxidant properties [12,13], among many others. This contrasts with the continued use of organic solvents and the numerous reasons for changes, including benefits to the environment, the health of handlers, and the quality of the bioactive extracted compounds. Organic solvents need a large volume of material, have greater resistance to mass transfer, and are associated with a possible degradation of the compounds of interest [14].

This scenario has stimulated research for new solvents as alternatives to replace organic solvents, such as natural deep eutectic solvents (NADESs) [15,16], which are ionic liquids formed by mixtures of available components, consisting of a hydrogen bond acceptor (quaternary ammonium salt, choline chloride—ChCl) and a hydrogen bond donor, such as natural primary metabolites, including sugars, sugar alcohols, organic acids, amino acids, and amines. These are characterized by extensive intermolecular interactions [17,18]. Different combinations of NADES components can produce adjustable affinity of the solvent for different compounds in plants. Therefore, not only are they greener and safer, NADESs could provide more alternatives with tunable efficiency and selectivity during the extraction process, which assists in customizing a more suitable extraction solvent system [19].

ChCl-NADESs are the most studied type of NADES for the isolation of biocompounds from natural resources. The physical and chemical properties of ChCl-NADESs resemble ionic liquids; therefore, ChCl-NADESs present high solubility and selectivity for polyphenolic compounds (PCs) [20]. Most ChCl-NADESs are environmentally friendly, non-toxic, and biodegradable organic compounds, which preserve the bioactivity of target compounds and are safe to use for food and other industrial chemical applications [16,18]. The properties of ChCl-NADESs are determined by their compounds and can be modified by altering the component species. Consequently, most NADESs are referred to as task-specific solvents [21]. The properties of ChCl-NADESs can be adjusted using different compounds as hydrogen bond donors (HBDs). Therefore, the characteristics of ChCl-NADESs, such as viscosity and phenolic acid polarity, can be made adequate to satisfy the requirements of the extraction equipment and match the attributes of the target compounds [22]. 

Recent studies have revealed the promising potential of NADESs for extracting anthocyanins from grape skin, wine lees, and *Catharanthus roseus* [23,24]. Binary NADESs composed of ChCl:glycerol or ChCl:citric acid have been shown to be as effective as 80% methanol in extracting anthocyanins from grape skin [24]. In addition, NADESs isolated anthocyanins, which reveals the potential industrial use of these solvents for food and other applications [23]. Moreover, NADESs have been recently shown to yield phenolic extracts of grape skin that have enhanced biological activity compared to organic solvents [25]. For this extraction, a customized ternary ChCl-NADES was used, comprising malic acid, urea and ChCl (2:1:2 molar ratio) to extract four phenolic acids, which exceeded the yield achieved using traditional organic solvents [26]. In addition, hydroxyl groups facilitate the excess of hydrogen bonds, thereby increasing the stability of NADESs [18]. The fact is that the acronym NADES has been used continuously as an increasingly broad concept, probably only highlighting how the solvent facilitates preparation, without the need for expensive purification procedures, which constitutes an additional justification for the marked increase in its use [16]. Despite the prospects for using NADESs for the extraction of bioactive compounds, they are being successfully used in other applications, such as carbon dioxide capture [27], drug synthesis [28], and also in determining analytical characteristics of compounds [29]. Considering all their properties, NADESs can be used for extraction or separation of polar and non-polar natural products from plants [25,30]. Knowledge regarding the characteristics of DESs (deep eutectic solvents), studies on their performance for extraction of biomolecules, and their effects compared to conventional extraction techniques complement the indications for changes from synthetic solvents to natural solvents. 

Because it is a simple, fast, efficient, and low-cost extraction procedure, ultrasound-assisted extraction has been increasingly used, not only for the extraction of biomolecules, but also for the quantification of other chemicals in foods. Moreover, it does not require long periods of time or high temperatures and pressures for preparation [31]. Therefore, the association of NADESs in ultrasound-assisted extraction of biomolecules warrants further attention. Ultrasound-assisted extraction (UAE) has been commonly used to improve the yield of phenolic compounds (PCs) and to shorten the time required to reach solid–liquid equilibrium during extraction of NADES ChCl. Yang et al. [32] used UAE to extract compounds from bioresources and reported a recovery efficiency (ER) rate of up to 100%. The extraction time of anthocyanins using a ChCl and citric acid mixture (2:1 molar ratio) as the ultrasound support media was only 10 min [33]. NADESs ChCl are considered very promising extraction solvents because their components form strong hydrogen bonds with target compounds. Hydrogen bonds prevent target molecules from being degraded, increasing extraction yield and stabilizing the produced extracts. Alam et al. [34] presented a comprehensive review of ChCl-based deep eutectic solvents as environmentally friendly extractants, and some studies on extraction processes using NADESs ChCl are summarized in Table 1.

The NADES ChCl:LA (choline chloride:lactic acid) was evaluated using three-dimensional (3D) response surface plots for interactions between three UAE-NADES extraction variables: time, molar ratio, and amount of water on the extraction efficiency of flavanones, chalcones, and total phenolic compounds [1], where an excellent adjustment of conditions was obtained for the extraction of bioactive compounds from inflorescence of *Helichrysum arenarium* L. The authors evaluated one of the eleven NADESs they prepared, and they emphasize that each one should be investigated to determine the optimal conditions for ultrasound-assisted extraction.

The aim of this study was to prepare different NADESs, determine their properties, and apply them in the extraction process of biomolecules from araza pulp using ultrasound.

## 2. Material and Methods

### 2.1. Preparation of the NADESs

**Figure 1:**
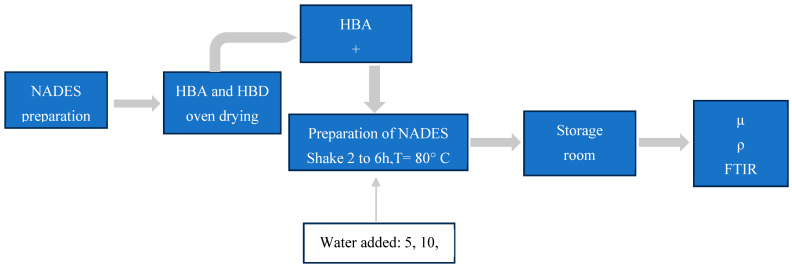
NADES preparation procedure.

All the solid reagents used in the preparation of the NADESs were dried in a vacuum oven (MA 030/12, Marconi, Piracicaba, São Paulo, Brazil), at 60 °C, for 24 h, before use [24]. The NADESs were obtained by using specific ratios of ChCl:malic acid (ChCl:MA), ChCl:fructose (ChCl:Fruc), ChCl:glucose (ChCl:Gluc), and ChCl:citric acid (ChCl:CA) [24]. All the NADESs were shaken in glass bottles (80 °C, 2 to 6 h), until a clear, homogeneous, and colorless liquid was formed. The water content added to the NADESs was determined following the procedure proposed by Dai et al. [38] (Table 2). The NADESs were prepared as shown in Figure 1.

### 2.2. Characterization of the NADESs

To evaluate whether the prepared solutions presented characteristic properties of NADESs, they underwent characterization.

#### 2.2.1. Fourier Transform Infra-Red (FTIR)

Each NADES was analyzed on a spectrophotometer FTIR Agilent Technologies (CARY 630 FTIR, Santa Clara, CA, USA), equipped with an ATR system with ZnSe crystal and adjusted for 32 scans, at a resolution of 4 cm^−1^. The spectral range was from 4000 cm^−1^ to 650 cm^−1^ with particular attention given to the region commonly referred to as “fingerprint” (2000 cm^−1^ to 700 cm^−1^) [4]. The response of interaction between the two components when mixed was investigated, including an examination of whether water has any influence on these vibrational bands.

#### 2.2.2. Raman Spectroscopy

The NADESs were characterized on a modulated Raman spectrometer (Horiba, IHR 320, Kyoto, Japan), using a Synapse charge-coupled device (CCD) for signal detection. The spectra were obtained with backscattered geometry. The samples were heated using a 785 nm laser (focused onto the sample with a 10X objective lens, focal length f = 10.5 mm, and numerical aperture NA = 0.35), with a power of ~0.2 mW, to achieve thermal equilibrium in the cross-section of the sample.

#### 2.2.3. pH Determination 

To measure the pH values of the NADES systems, 0.5 mol dm^−3^ aqueous NADES solutions were prepared as described by Skulcova et al. [39]. The NADES samples were weighed and dissolved in a corresponding volume of deionized water. The freshly prepared solutions were kept at 25 ± 0.2 °C for 30 min. The pH values were determined using a digital pH meter (0–14), previously calibrated (Instrutherm, PH-1900, São Paulo, Brazil) with standard solutions of pH 4.0 and 7.0. The measurements were performed at 25 °C.

#### 2.2.4. Viscosity and Density

The rheological properties of the NADESs without water were characterized on a Brookfield Rheometer (Model R/S Plus-SST, Middleboro, MA, USA) with a C-50 plate cone system, using a bath (Lauda RE 206 ECOLINE, Delran, NJ, USA), at 40 ± 0.1 °C. Data were obtained for each NADES sample using the control rate (CR) and ramp method with a shear ramp ranging from 0.1 to 300 s^−1^. The measurements were performed in triplicate for each sample.

The density of the NADESs was evaluated using a hydrometer (Gehaka, DSL-900, São Paulo, Brazil) at 25 ± 0.2 °C. The measurements were performed in triplicate and presented in g mL^−1^.

### 2.3. Determinations of the Characteristics of the Raw Material

The physicochemical characterization of the fresh araza pulp was carried out: moisture (nº 925.10), protein (nº 920.87), pH (nº 981.12), titratable acidity (nº 942.15), and soluble solids, according to methodologies described by the AOAC [40]. The determination of the content of phenolics in araza pulp was carried out using the Folin–Ciocalteu method [41]. The antioxidant activity was determined by ABTS and DPPH radical scavenging assays, as described by Kuskoski et al. [42] and Mensor et al. [43], respectively.

### 2.4. Sample Preparation

The araza fruits were harvested in March 2021, at the coordinates 01°07′44″, 47°37′12″, and processed at the Physical Measurements Laboratory at the Federal University of Pará, Belém-Pará, Brazil. They were sanitized, peeled, homogenized, and their pulp was freeze-dried and stored at −18 °C until analysis.

### 2.5. Process of Extraction

**Figure 2:**
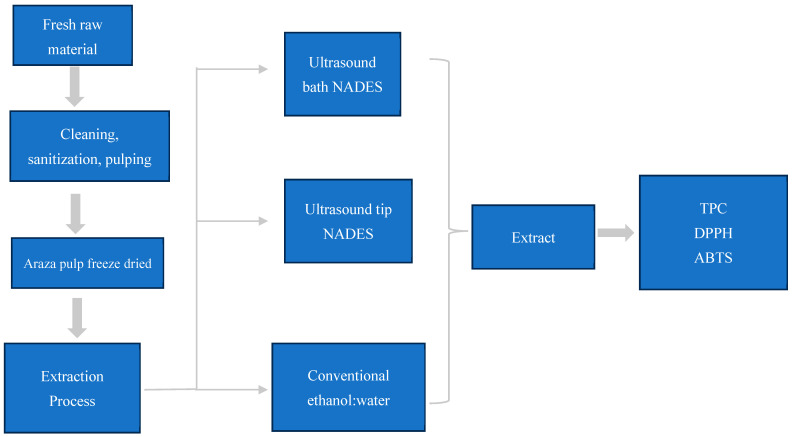
Extraction process, using NADESs and conventional solvents.

Phenolic compounds from freeze-dried araza pulp were extracted using NADESs. Two types of ultrasounds were used to evaluate the extraction process, namely, bath and tip ultrasound. Ethanol:water (70:30 *v*/*v*) was used as a reference method from organic solvent extraction (Figure 2). 

#### 2.5.1. Extraction by Using NADESs

NADESs consist of a mixture of a hydrogen bond acceptor and a hydrogen bond donor, formulating a eutectic mixture. They offer particular advantages due to their physicochemical properties, such as adjustable surface tension and viscosity. NADESs have been suggested for the extraction of phenolic compounds as alternatives to conventional organic solvents, offering both enhanced extraction efficiency and quality of the extracts. The combination of innovative extraction techniques using NADESs for the extraction of phenolic compounds from araza pulp was adopted in this study.

##### Ultrasonic Bath-Assisted Extraction

The freeze-dried sample of araza pulp (1 g) was used for extraction. It was placed in conical tubes (Falcon) with 20 mL of the NADES, then prepared and subjected to extraction in an ultrasonic bath (Schuster, L200, Santa Maria, Rio Grande do Sul, Brazil). The extraction parameters were 30 °C, time of 5 min, ultrasound frequency of 35,000 Hz, 170 W. The extraction conditions were according to the methodology described by Paniċ et al. [33]. The extracts were centrifuged at 1008× *g* for 10 min and the supernatant was collected and subjected to analysis of total phenolic compounds and antioxidant activity [44].

##### NADES Ultrasound Tip Extraction

A Qsonica sonicator Q700 (Newtown, CT, USA) was used, following the same procedure described in the previous extraction. The extraction parameters were 30 °C, time of 5 min, ultrasound amplitude of 50, and standard 1/2″ diameter probe with replaceable tip. The extraction conditions were according to the methodology described by Paniċ et al. [33]. 

The extracts were centrifuged at 1008× *g* for 10 min and the supernatant was collected and subjected to analysis of total phenolic compounds and antioxidant activity [44].

#### 2.5.2. Conventional Extraction

In a conventional extraction, ethanol:water (70:30 *v*/*v*) was used as solvent under the same experimental conditions used in extractions with the NADESs (T = 30 °C, 1:20) according to the methodology described by Chanioti, Katsouli, and Tzia (2021) [45].

#### 2.5.3. Yield of the Process

The yield of the NADES extraction process was evaluated by the experimental procedure repetition using ethanol:water (70:30 *v*/*v*) as a conventional solvent [46] (Equation (1)).

(1) $$Y_{process}=\frac{{TPC}_{NADES}}{{TPC}_{Ethanol}}\times 100$$

where *TPC* is total phenolic content in the extract. 

### 2.6. Characterization of the Extract 

#### 2.6.1. Total Phenolic Compounds (TPC)

The content of total phenolic compounds (TPC) was determined using the Folin–Ciocalteu method [41], and the absorbance was measured at 760 nm using a UV–visible spectrophotometer after 5 min of reaction in a water bath at 50 °C. The total phenol content was evaluated using a calibration curve, and the results were expressed as mg of gallic acid equivalent (GAE) per g of sample dry weight (mg GAEg^−1^).

#### 2.6.2. Antioxidant Activity

The antioxidant activity analysis using the ABTS method was performed as described by Kuskoski et al. [42], and the absorbance readings were performed at 754 nm. A standard Trolox curve was employed, and the results were expressed in µmolL^−1^ of Trolox g^−1^.

The DPPH radical assay was determined as described by Mensor et al. [43]; absorbance readings were performed at 520 nm. A standard Trolox curve was employed, and the results were expressed as EC50% in µmolL^−1^ Trolox g^−1^ sample.

## 3. Results and Discussion

### 3.1. Characterization of the NADESs 

#### 3.1.1. Density and Viscosity

It is well-established that density and viscosity are important properties for the utilization of solvents in extraction processes. Viscosity and density measurements were carried out to evaluate the feasibility of using different NADESs without the addition of water in the study of the extraction process (Table 3). 

Due to the higher viscosity and density values noted for the NADESs without the addition of water, in comparison to traditional organic solvents, it was observed that it would be necessary to add water to the NADES formulations so that they could be used as solvents in the extraction process.

Although the addition of water to NADESs offers advantages, its utilization requires careful analysis. Gabriele et al. [48] observed a break in hydrogen bonds from 50% (*v/v*) due to the utilization of water. High water concentrations (>50%) also resulted in a decrease in anthocyanin extraction yield in studies conducted by Panić et al. [33] and Bosiljkov et al. [49].

In this study, the addition of 5%, 10%, 20%, 30%, and 40% of water to different NADES formulations was investigated.

#### 3.1.2. Fourier Transform Infrared Spectroscopy (FTIR)

**Figure 3:**
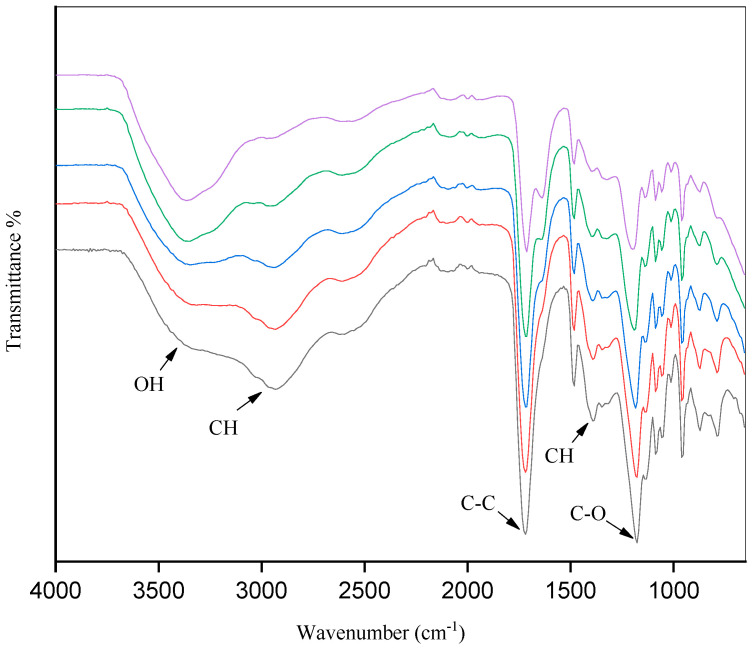
FTIR Spectra of NADES ChCl:CA containing varying proportions of water: 5% water (black line); 10% water (red line); 20% water (blue line); 30% water (green line); 40% water (violet line).

According to the FTIR spectrum obtained for the ChCl:CA (Figure 3), the most notable change was the increase in the intensity of the region corresponding to OH as the percentage of water was increased, with no alterations detected in the other vibrational modes. The extraction of biomolecules by the NADESs is influenced by both the water content and the nature of each compound evaluated, which are key factors for the extractability and bioactive properties observed [16,50,51]. 

**Figure 4:**
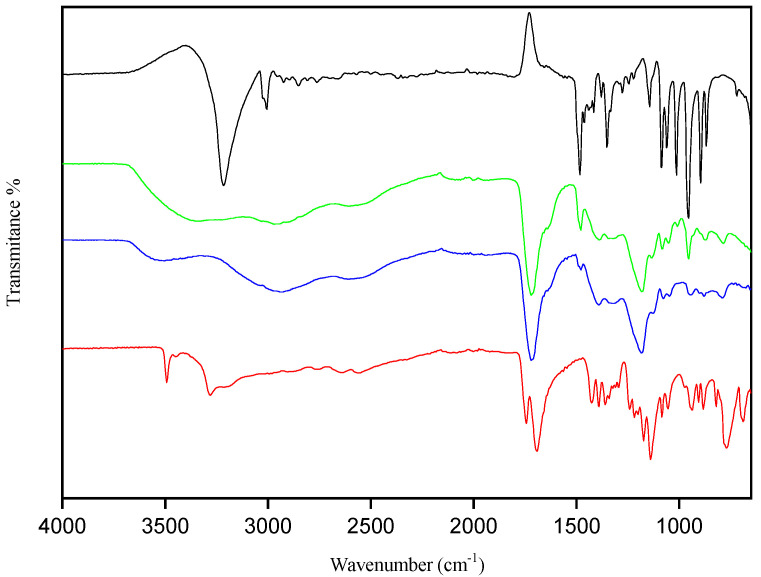
FTIR spectra: ChCl (black line), ChCl:CA (green line), ChCl:CA:water 40% (blue line), Ca (red line).

The supramolecular structures of the NADESs were investigated by FTIR spectroscopy to evaluate the interactions between the hydrogen bond acceptor (ChCl) and the other reactants (hydrogen bond donors). The FTIR spectra of the pure components and the NADES ChCl:CA, both in the presence and absence of water, are show in Figure 4.

An increase in intensity was observed in the OH region of the spectrum as the percentage of water increased, while the vibrational modes remained preserved (Figure 4). Pires et al. [46] reported similar behavior in the characterization of different NADESs based on lactic acid and glycine.

The primary indication of hydrogen bond formation between the hydrogen bond acceptor and donor is the changes observed in the stretching vibration of the NADESs at the wave number of 3300 cm^−1^ [48,49]. These results confirm the synthesis of the NADES solvent with the increase in and strengthening of hydrogen bonds.

The O–H stretching vibration in the MA spectrum (Figure S1c) is confirmed by the band at 3435 cm^−1^. The bands at 1739 cm^−1^ and 1681 cm^−1^ are attributed to C–O stretching and H–O–H scissor vibrations, respectively. The band at 1359 cm^−1^ is attributed to C–H bending vibrations. The characteristic bands of MA are also described by Dai et al. [38], who characterized the mixture of proline and malic acid.

The bands at 600 and 1500 cm^−1^ comprise the fingerprint region of the molecule [50]. The bands at 800–600 cm^−1^ are further evidence of the contribution of salts to the NADES structure, representing halide ions (Br and Cl) [51].

The citric acid band at 3362 cm^−1^ is relative to the O–H vibrations, as shown in Figure 2. According to Silva et al. [52], who also analyzed citric acid, these vibrations of the free OH band at the wavelengths between 3493 cm^−1^ and 3445 cm^−1^ can be attributed to the water content, as a monohydrate acid. Bands at 1743 cm^−1^ and 1691 cm^−1^ are attributed to RCOOR stretching and C–C scissor vibrations, respectively. C–H bending vibrations are observed at 1389 cm^−1^, while peaks at 1240 cm^−1^ and 1139 cm^−1^ correspond to C–O vibrations.

The FTIR spectrum of ChCl shows a broad band at 3415 cm^−1^ attributed to O–H vibration. Bands that are barely visible at 2954 cm^−1^ and 2923 cm^−1^ correspond to C–H vibrations. The relatively broad band at 1648 cm^−1^ corresponds to the H–O–H scissor vibrations in the water molecule. The C–H bending vibrations are detected as a signal at 1482 cm^−1^, and bands at 1084 cm^−1^ and 1059 cm^−1^ are attributed to C–O stretching vibrations and C–O–H bending vibrations.

The signals at 3392 cm^−1^ and 1639 cm^−1^ of the fructose and glucose (Figure S1a,b) are attributed to νOH and δOH groups, respectively. The deformation modes C–C–H and C–O–H are located close to 1400 cm^−1^ [53].

The bands between 3700 cm^−1^ and 3000 cm^−1^ of ChCl:Fruc containing 40% water and ChCl:Gluc containing 40% water (Figure S1a,b), which are associated with the water, are wider than in the anhydrous mixture. It is indicated that hydrogen bonds were formed between coordinated water and ChCl (HO–H and OH–Cl), and these bonds were more intense with the addition of water than in the anhydrous formulations ChCl:Fruc and ChCl:Gluc, respectively.

#### 3.1.3. Raman Spectroscopy

**Figure 5:**
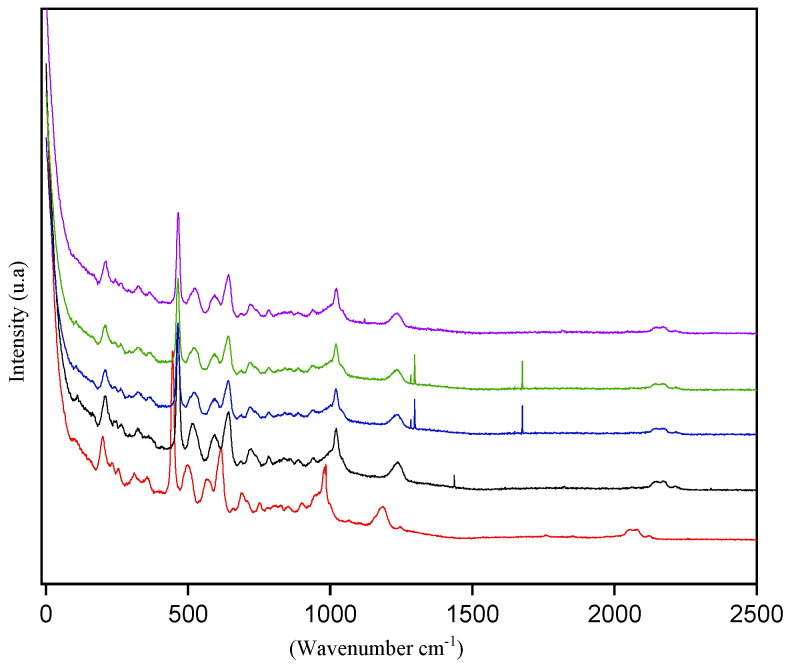
Raman spectra of the NADESs ChCl:CA with different proportions of water. Water content: 5% (red line); 10% (black line); 20% (blue line); 30% (green line); 40% (violet line).

Vibrational spectroscopy techniques can provide information relevant to solvent–solvent interactions. The structural characteristics of the NADESs were studied to gain a deeper understanding of the interactions that occur between a hydrogen bond acceptor (ChCl) and the other reactants (donors). Raman spectra of the NADES ChCl:CA containing different water concentrations did not change (Figure 5), indicating that water did not influence the vibrational behavior.

Molecular dynamics simulations have also been employed to investigate the molecular interactions of NADESs and their aqueous solutions [54,55]. The results of the simulation revealed that when water is added in small quantities, it is adsorbed onto the NADESs, and the bonds in the liquid transactions do not change up to 50% of the water fraction [55]. Similar results were found for all the NADESs studied (Figure S2).

**Figure 6:**
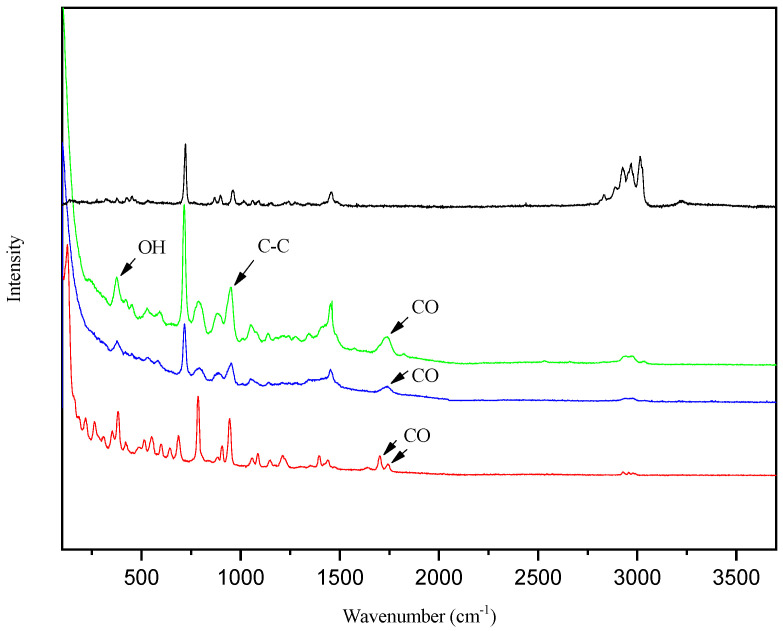
Raman spectra of ChCl (black line), ChCl:CA (green line), ChCl:CA:water 40% (blue line), CA (red line).

Figure 6 presents the Raman spectra of the NADESs ChCl:CA in solid state and in solutions, measured from 100 cm^−1^ to 4000 cm^−1^; there are narrow vibration bands between 400 cm^−1^ and 1500 cm^−1^. 

Two characteristic vibrational bands of solid citric acid are visible (1701 cm^−1^ and 1741 cm^−1^), corresponding to symmetric C–O stretching. According to Bichara et al. [56], these features should be relative to other C–O stretching modes, confirming the presence of dimers in the solid state. In solutions, these bands are intensified and shifted to 1736 cm^−1^ and 1740 cm^−1^, indicating the formation of hydrogen bonds between the components. Moreover, in the shorter wavelength region (381 cm^−1^), OH torsional modes are expected and these modes are also affected by H bonding. The bands observed in the IR spectrum at 945 cm^−1^ are attributed to C–C stretching modes.

In the Raman spectrum of ChCl:MA (Figure S3c), the vibrational band present at 1458 cm^−1^ in the solid choline chloride, which corresponds to the deformation vibration of the C–H bond, is also observed in the solutions at 1454 cm^−1^ and 1455 cm^−1^, respectively (Figure S3c). Another characteristic vibrational band of solid malic acid is visible at 1655 cm^−1^, and corresponds to symmetric C–O stretching. The presence of this band is also described by Baranska et al. [57]. 

In the Raman spectrum of solid ChCl and that of NADES ChCl:Fruc (Figure S3a), two vibrational bands of these solutions correspond to the vibrational characteristics of ChCl molecules in the solid state (960 cm^−1^ and 1458 cm^−1^), which originated from intramolecular vibrations, such as stretching vibration of the CN group and deformation of the C—O–H bond. Additionally, two vibrational bands of these solutions refer to the characteristic vibrations of the fructose molecules in the solid state at 527 cm^−1^ and 626 cm^−1^, respectively, and are attributed by Ruggiero et al. [58] for bending modes δ(C–C–O).

In the Raman spectrum of glucose in the solid state and that of the NADES ChCl:Gluc (Figure S4b), two bands (521 cm^−1^ and 1133 cm^−1^) of the glucose solutions refer to the vibrational characteristics of glucose molecules in the solid state (542 cm^−1^ and 1121 cm^−1^), which originate from intramolecular vibrations, such as deformation of the C–C–O and C–O–H groups. The Raman spectra of fructose and glucose reported in this study (Figure S4a,b) are in agreement with those presented by other authors [58,59].

#### 3.1.4. pH Determination

Table 4 presents the pH values observed for the NADESs containing 20% and 40% water. The characteristics of the hydrogen bond donor determine the acidity of the mixture obtained.

Therefore, the nature of the hydrogen bond donor impacts acidity and glucose-based NADESs are less acidic than fructose-based NADESs when combined with choline chloride. Environmentally friendly solvents are classified into two groups: acidic (pH values of 2.0 to 3.0) and neutral (pH values of 6.0 to 7.0) [60]. The NADESs investigated in this study were classified into two groups: acidic (pH ranging from 2.34 to 2.45) and close to neutral (pH ranging from 7.10 to 7.38). pH measurements were conducted only for the NADESs with 20% and 40% water, as these concentrations were utilized in the study of the extraction process due to the high viscosity and density presented by other NADESs. Given the methodology used to measure pH, which involves diluting a quantity of the NADES with water, there was no variation in pH between the two NADESs measured.

pH has an essential impact on chemical reactions. The interference of pH is important for applications of NADESs in catalysis [61], biochemical reactions or in the treatment of metals [18,62]. Hayyan et al. [63] studied several types of DESs based on fructose and ChCl. The results indicated that as hydrogen bond donor content decreased, the pH value also decreased. However, higher fructose content in the formulation leads to higher pH. Hayyan et al. [63] reported pH values as a function of temperature for NADESs based on ChCl and glucose, with the pH ranging from 6.03 to 7.11. In the present study, the NADESs based on fructose and on glucose have a pH around 7, while those based on citric acid and on malic acid have a pH below 2.5, indicating a direct interference of the type of hydrogen donor in the pH of the NADES formulations.

The second group comprises organic acid-based NADESs, whose structure affects pH values, ranging from 2.34 to 2.45 at (25 ± 2 °C). This study of pH behaviors demonstrates that the hydrogen bond donor strongly affects the resulting pH, thus dictating the acidity of the mixture obtained [39]. Generally, the acidity and basicity of DESs are governed by the acidity/basicity constants of the donor and acceptor materials used, as well as their combination. This fact was also demonstrated by Kareem et al. [64], who determined that the type of donor significantly affected the acidity of NADESs.

After preliminary tests, the NADESs made with ChCl, citric acid, malic acid, glucose, and fructose, each containing 20% and 40% water, were selected to be used in the study of the extraction process of bioactive compounds from araza pulp.

### 3.2. Properties of the Raw Material 

The fresh araza pulp had a moisture content of 95.11 ± 0.06 g 100 g^−1^, protein content of 0.20 ± 0.01 g 100 g^−1^, total soluble solids of 2.5 ± 0.00 °Brix, pH of 2.71 ± 0.01, and acidity of 1.11 ± 0.02 (citric acid g 100 mL^−1^). Araza is a fruit with high moisture content, a common characteristic among the araza species, making it more perishable compared to fruits from the Amazon region, such as abiu, yellow mangosteen, and achachairu [60,65]. The acidity, expressed in terms of citric acid, is close to the values found for fruits such as acerola and soursop [60]. The total phenolic content (TPC) in the araza pulp was 484.25 mg GAE100 g^−1^, which is higher than the values reported by Araujo et al. [66] (115 mg GAE 100 g^−1^) and by Neri-Numa et al. (184.08 mg GAE 100 g^−1^) [2] for the *Eugenia stipitata* McVaugh Myrtaceae. Given its wild cultivation and the various growing conditions across different regions of Brazil, the araza fruit, which typically weighs between 30 g and 300 g, can exhibit a wide variability in its composition of bioactive compounds. Araujo et al. [66] identified 15 compounds in the pulp of *Eugenia stipitata*, belonging to different classes, such as organic acids, phenolic acids, and flavonoids. Among the compounds present in araza are apigenin hexoside, cinnamic acid, caffeoyl tartaric acid, caffeoyl hexose, caffeoyl methylquinic acid, coumaroyl tartaric acid, fertaric acid, gallic acid hexoside, methylapigeninhexoside, catechin dihexoside, gallocatechin, luteolin hexoside, and luteolin malonyldihexoside. These compounds have various beneficial properties including antimicrobial, anti-inflammatory, anti-obesity, antioxidant, and prebiotic effects [67].

The results obtained for the ABTS and DPPH assays in this study were 1.18 ± 0.13 µmolL^−1^ Trolox g^−1^, and 72.78 ± 1.69 µmolL^−1^ Trolox g^−1^, respectively. The values noted were higher than those reported by Neri-Numa et al. (2013) and Araujo et al. (2021) [2,66].

#### Total Phenolic Content and Antioxidant Activity

The results of TPC, DPPH, and ABTS content in araza pulp (*Eugenia stipitata*), obtained from extracts using different processes with NADESs and with ethanol, are presented in Table 5.

In the study of the bath extraction process, increasing the water content in the NADESs from 20% to 40% promoted a decrease in the efficiency of TPC extraction for NADES formed by sugars (fructose and glucose) and an increase for the NADESs formed by organic acids (malic and citric).

For the tip extraction process, increasing the water content was crucial for the feasibility of the study. With the NADESs containing 20% water, it was not possible to evaluate the extracts obtained in a spectrophotometer. The use of tip ultrasound to assist the extraction process, even over a short duration, generated a sufficient increase in temperature. In the case of the NADESs containing 20% water, this increase in temperature caused the darkening of the extract and the “caramelization” of the compounds of araza pulp. This effect caused the extract to become dark and cloudy, preventing accurate spectrophotometer readings. In the case of the NADESs containing 40% water, this effect was not observed due to their higher water concentration and lower viscosity. The results indicate that TPC extraction using tip ultrasound with 40% water was more efficient, yielding higher values for the NADESs formulated with malic acid and with citric acid.

The efficiency of TPC for the different extraction methods was obtained by ethanol > tip ultrasound > bath ultrasound. For the tip ultrasound, the TPC content was ChCl:CA > ChCl:MA > ChCl:Fruc > ChCl:Gluc, with values between 23.96 and 273.40 mg GAE 100 g^−1^. For the extractions in an ultrasound bath, the water content influenced the efficiency of TPC extraction, and the best results were for ChCl:Fruc > ChCl:Gluc with 20% water and ChCl:CA > ChCl:MA with 40% water. This highlights the crucial role of selecting the hydrogen acceptor, whether it be sugar or organic acid, in the extraction of bioactive compounds from the same food matrix.

The extraction obtained with the NADES ChCl:CA (1:1) containing 40% water yielded approximately 84% of the TPC from the araza pulp, compared to ethanol extraction. Studies that evaluated the extraction of biomolecules from different processes and plant matrices, using NADESs based on ChCl, obtained similar extraction yields (85%, 90%, and 84%) [18,37]. 

These results can be attributed to the composition of araza pulp, where the NADESs with lower pH (more acidic) obtained the best results for extraction yield. Previously reported, acid-based NADESs provide superior extraction of TPC from plant tissues compared to sugar-based NADESs [63]. In their research, Popovic et al. [35] compared different extraction methods using NADESs containing ChCl:MA and ChCl:Fruc and observed similar performance between the NADESs based on acids and based on sugars. The positive effect of these systems on the extraction of anthocyanins has been previously described and is attributed to the acidity of these solvents [16,23].

In a study conducted by Bosiljkov et al. [47], NADESs based on acids and sugars also showed differences in performance, where the NADESs based on organic acids showed higher efficiency than those based on sugars. The difference in efficiency of the various NADES systems is also attributed to the differences in polarity. Among the NADESs tested in this study, those based on organic acid were found to be more polar than those based on sugars and polyalcohols [18]. 

The DPPH and ABTS values of the obtained extracts varied widely between extraction methods, ranging from 1.18 µmolL^−1^ to 31.55 µmolL^−1^, and from 13.85 troloxg^−1^ to 204.9 troloxg^−1^ of sample, respectively. Higher values were obtained in tip ultrasound extraction by ChCl:CA > ChCl:MA > ChCl:Fruc > ChCl:Gluc NADESs containing 40% water. A positive correlation was observed between antioxidant properties (DPPH and ABTS) and the content of TPC.

In general, NADESs can serve as good replacements for organic solvents in the extraction of phenolic compounds, with at least one NADES showing an extraction yield equivalent to that of ethanol [18,64]. In this study, at least one NADES showed an extraction yield equivalent to that of ethanol:water (70:30 *v*/*v*), consistent with prior research conducted with these solvents.

The lower values found in ultrasound in a water bath may be attributed to the high concentration of volatile compounds [33], which can be lost during the extraction process due to the open equipment configuration (water bath) and exposure to the environment. Even with the tube closed, small losses of volatile compounds may occur due to contact with the external environment. This phenomenon was not observed in the tip ultrasound extraction, where the process takes place in a closed chamber without contact with the external environment. Therefore, the NADES with acidic range of pH value (2.34) using citric acid as hydrogen donor was more efficient than the NADES with pH close to 7.0, or even the one with malic acid, despite malic acid being within the pH range of citric acid for phenolic compound extraction. These results prove that the pH of DESs influences the extraction of biomolecules, with positive results obtained in ultrasound extraction with a tip.

Neutral solvents were found to be less efficient compared to acidic solvents [33,39]. In araza pulp, using organic solvents for extraction yielded organic acids and flavonoids with polar characteristics. This justifies the prevalence of hydrogen interactions in acidic NADESs, resulting in greater efficiency. These findings are consistent with the results previously observed in the FTIR spectra, which demonstrate better coordination for the extraction of materials with the characteristics of the biomolecules of araza pulp. The performance of the studied solvents, according to their solvation properties, obtained with the different formulations, resulted in different effects on the extraction of phenolic compounds from araza pulp. Choline chloride, with its 14 hydrogen atoms, and citric acid and malic acid as hydrogen donors, contribute to increasing the polar characteristics in the medium.

Considering the important conclusions regarding the suitability of formulations and the compounds to be extracted, the initiatives aimed at developing and utilizing new solvents, particularly those involving other sources of biomolecules, hold promise. However, among ionic liquids (ILs), deep eutectic solvents and NADESs, which are low-cost and environmentally friendly, are the most attractive in different applications [16].

## 4. Conclusions

NADESs with no addition of water presented high viscosity and density and were not viable for studying the extraction process. The Raman and FTIR spectra for all the NADESs that were prepared showed differences in intensity as the proportion of water increased, while maintaining consistent behavior. The pH of the NADESs ranged from 2.34 to 7.38. The more acidic the pH of NADES, the better the TPC extraction yield. Only the NADESs containing 40% water showed suitable properties for use in the extraction of bioactive compounds from araza pulp in an ultrasound bath and tip ultrasound. The NADES ChCl:CA (1:1) containing 40% water showed 84% yield when compared to extraction with ethanol, indicating its potential for application as a solvent in the food industry. The results indicate that NADESs are versatile and environmentally friendly solvents capable of extracting various bioactive compounds present in foods. The key to successful extraction lies in understanding the compound to be extracted and selecting the NADESs with the appropriate properties.

## Figures and Tables

**Table 1:** Phenolic compounds extracted from different biological sources using hydrogen acceptor choline chloride-based deep eutectic solvents.

Hydrogen Donor	Source	Bioactive Compound	Reference
MA	*Carya cathayensis* Sarg	Polyphenols	[19]
Fruc, MA	Cherry pomace	Polyphenols	[35]
CA	Grape and olive pomace	Polyphenols	[33]
Gluc, Fruc	*Lippia citriodora*	Polyphenols	[36]
CA, Gluc, MA	*C. cajan* leaves	Polyphenols	[37]
LA, MA, Gluc, Fruc	*Helichrysum arenarium* L.	Polyphenols	[1]

Fruc: fructose; Gluc: glucose; MA: malic acid; CA: citric acid; LA: lactic acid.

**Table 2:** Molar ratio used for the preparation of the NADESs.

Hydrogen Bond Acceptor (HBA)	Hydrogen Bond Donor (HBD)	Molar Ratio	Water * (%)
Choline chloride	Fructose	5:2	0–40
Choline chloride	Glucose	5:2	0–40
Choline chloride	Malic acid	1:1	0–40
Choline chloride	Citric acid	1:1	0–40

* Water added to the NADESs: 0%, 5%, 10%, 20%, 30%, and 40%.

**Table 3:** Viscosity (µ) and density (ρ) properties for the NADESs at 40 °C.

NADESs	ρ (g mL^−1^)	µ Pa s
ChCl:Fruc	1.277 ± 0.054	0.24777
ChCl:Gluc	1.234 ± 0.002	0.2723
ChCl:MA	1.281 ± 0.003	0.09991
ChCl:CA	1.375 ± 0.020	0.46921
* Ethanol	0.783	0.0008

The data above show mean of triplicate ± standard deviation. (*) CRC Handbook of Chemistry and Physics (2023) [47].

**Table 4:** pH of the NADESs.

NADESs *	pH
ChCl:Fruc	7.10
ChCl:Gluc	7.38
ChCl:MA	2.45
ChCl:CA	2.34

* NADESs containing 20% and 40% water.

**Table 5:** Total phenolic content (TPC) and antioxidant activity of extracts obtained with the different NADESs containing 20% and 40% water.

NADES	Ultrasound Bath			Ultrasound Tip		
	TPC	ABTS	DPPH	TPC	ABTS	DPPH
NADESs 20%						
ChCl:Fruc	161.99 ± 3.06	10.2 ± 0.20	73.8 ± 8.18	ND	ND	ND
ChCl:Gluc	88.51 ± 3.48	10.8 ± 0.26	78.9 ± 5.56	ND	ND	ND
ChCl:MA	177.43 ± 1.07	45.93 ± 0.75	148.9 ± 1.49	ND	ND	ND
ChCl:CA	173.15 ± 2.73	29.37 ± 0.73	96.9 ± 2.0	ND	ND	ND
NADESs 40%						
ChCl:Fruc	48.50 ± 0.83	13.85 ± 1.4	1.18 ± 0.15	49.61 ± 0.49	157.06 ± 8.18	25.19 ± 0.40
ChCl:Gluc	17.40 ± 3.66	14.18 ± 2.1	1.23 ± 0.12	23.96 ± 4.83	160.0 ± 5.56	15.66 ± 0.58
ChCl:MA	206.81 ± 1.83	29.14 ± 1.8	8.72 ± 0.34	183.76 ± 7.73	173.56 ± 9.68	29.39 ± 0.43
ChCl:CA	225.26 ± 2.75	61.10 ± 0.9	14.20 ± 0.20	273.40 ± 13.09	204.9 ± 12.89	31.55 ± 0.43
Conventional						
Ethanol:water (70:30)	325.19 ± 7.38	291.31 ± 8.4	12.00 ± 0.4			
Freeze-dried pulp						
Araza pulp	484.25 ± 10.99	72.78 ± 1.69	1.18 ± 0.13			

The data above show the mean from the triplicate ± standard deviation. |The data express TPC—mg GAE100 g^−1^; DPPH and ABTS—µmolL^−1^ and Trolox g^−1^.

## Data Availability

The original contributions presented in the study are included in the article/Supplementary Material, further inquiries can be directed to the corresponding author.

## Supplementary Materials

The following supporting information can be downloaded at: https://www.mdpi.com/article/10.3390/foods13131983/s1, Figure S1: Spectra (FTIR) NADES with different water content. Water 5% (black line); water 10% (red line); water 20% (blue line); water 30% (green line); water 40% (violet); Figure S2: Spectra FTIR chloride choline (black line); NADES (green line); 40% water NADES (blue line); fructose (a), glucose (b) and malic acid (c) (red line); Figure S3: Spectra (RAMAN) NADES with different water content. Water 5% (black line); water 10% (red line); water 20% (blue line); water 30% (green line); water 40% (violet); Figure S4: Spectra RAMAN chloride choline (black line); NADES (green line); 40% water NADES (blue line); fructose (a), glucose (b) and malic acid (c) (red line).
