# Supplementary material for: Preparation and Characterization of Natural Deep Eutectic Solvents (NADESs): Application in the Extraction of Phenolic Compounds from Araza Pulp (Eugenia stipitata)
Source: Foods. 2024 Jun 24;13(13):1983. doi: 10.3390/foods13131983 (PMC11241137; doi:10.3390/foods13131983)
Supplement: Supplementary file 1 [file foods-13-01983-s001.zip › foods-3009785-supplementary.pdf]

Preparation and Characterization of Natural Deep Eutectic Solvents (NADESs): Application in the Extraction of Phenolic Compounds from Araza Pulp (*Eugenia stipitata*)

Yasmin Caroline Nóvoa Sakurai [1], Ianê Valente Pires [1], Nelson Rosa Ferreira [1], Sanclayton Geraldo Carneiro Moreira [2], Luiza Helena Meller da Silva [1,*] and Antonio Manoel da Cruz Rodrigues [1]

[1] Programa de Pós-Graduação em Ciência e Tecnologia de Alimentos, Universidade Federal do Pará, Rua Augusto Correa S/N, Guamá, Belém 66075-900, PA, Brazil; yasmin_novoa@hotmail.com (Y.C.N.S.); iane_valente@hotmail.com (I.V.P.); nelson.ufpa@gmail.com (N.R.F.); amcr@ufpa.br (A.M.d.C.R.)

[2] Instituto de Ciências Exatas e Naturais (ICEN), Universidade Federal do Pará, Rua Augusto Correa S/N, Guamá, Belém 66075-900, PA, Brazil; sanclay@ufpa.br

* Correspondence: lhmeller@ufpa.br; Tel.: +55-91-991-651-264

Supplementary material

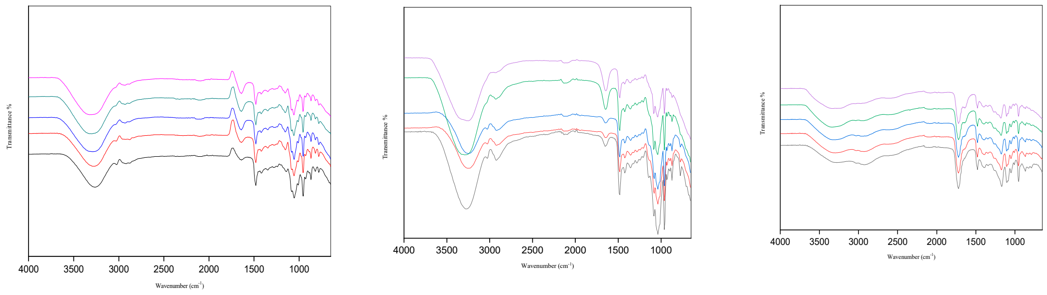

(a) ChCl:Fruc (b) ChCl:Gluc (c) ChCl:MA

Figure S1. Spectra (FTIR) NADES with different water content. Water 5% (black line); water 10% (red line); water 20% (blue line); water 30% (green line); water 40% (violet)

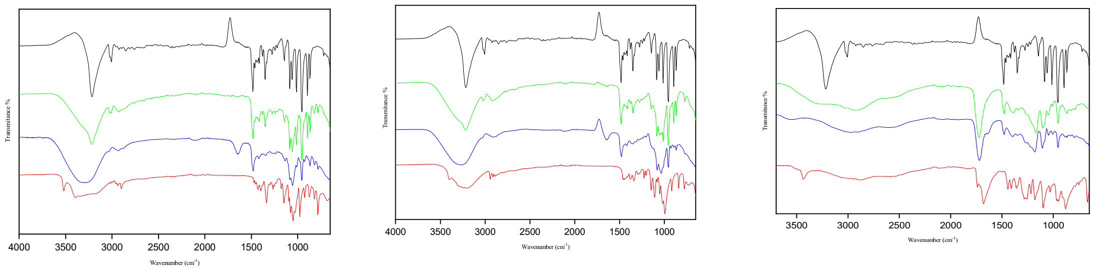

(a) ChCl:Fruc (b) ChCl:Gluc (c) ChCl:MA

Figure S2. Spectra FTIR chloride choline (black line); NADES (green line); 40% water NADES (blue line); fructose (a), glucose (b) and malic acid (c) (red line).

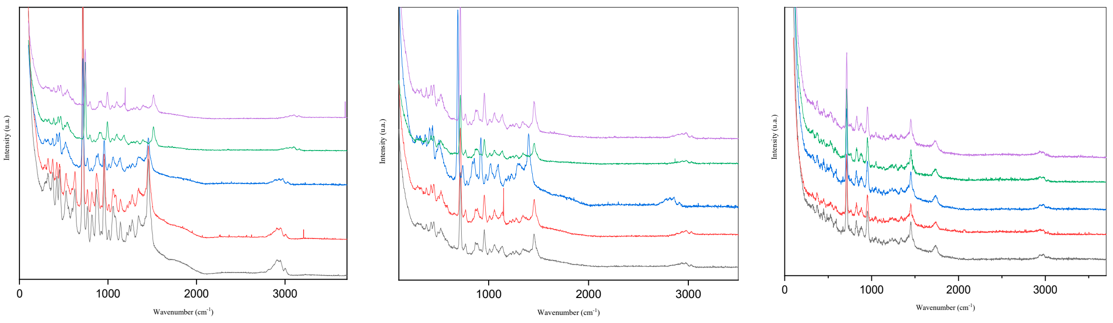

(a) ChCl:Fruc (b) ChCL:Gluc (c) ChCl:MA

Figure S3. Spectra (RAMAN) NADES with different water content. Water 5% (black line); water 10% (red line); water 20% (blue line); water 30% (green line); water 40% (violet).

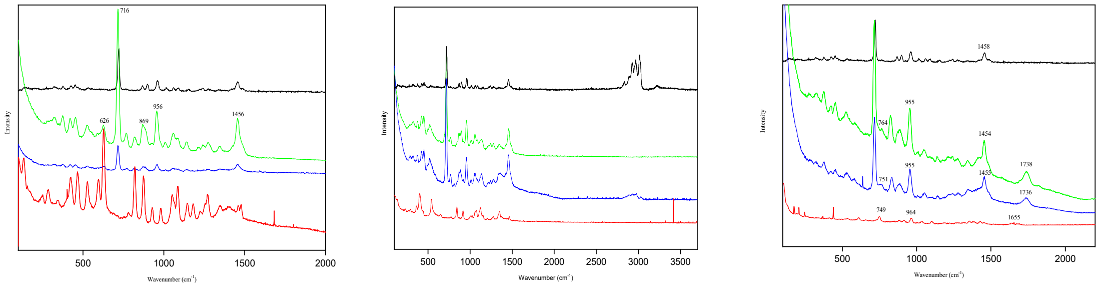

(a) ChCl:Fruc (b) ChCl:Gluc (c) ChCl:MA

Figure S4. Spectra RAMAN chloride choline (black line); NADES (green line); 40% water NADES (blue line); fructose (a), glucose (b) and malic acid (c) (red line).
